# Inhibition by Marine Algae of Chikungunya Virus Isolated From Patients in a Recent Disease Outbreak in Rio de Janeiro

**DOI:** 10.3389/fmicb.2019.02426

**Published:** 2019-10-24

**Authors:** Claudio Cesar Cirne-Santos, Caroline de Souza Barros, Caio Cesar Richter Nogueira, Renata Campos Azevedo, Kristie Aimi Yamamoto, Guilherme Louzada Silva Meira, Zilton Farias Meira de Vasconcelos, Norman Arthur Ratcliffe, Valéria Laneuville Teixeira, Jonas Schmidt-Chanasit, Davis Fernandes Ferreira, Izabel Christina Nunes de Palmer Paixão

**Affiliations:** ^1^Laboratório de Virologia Molecular e Biotecnologia Marinha, Programa de Pós-graduação em Ciências e Biotecnologia, Departamento de Biologia Celular e Molecular, Instituto de Biologia, Universidade Federal Fluminense, Niterói, Brazil; ^2^Departamento de Ensino, Curso de Farmácia na Universidade Salgado de Oliveira, Niterói, Brazil; ^3^Laboratório de Produtos Naturais de Algas Marinhas (ALGAMAR), Departamento de Biologia Marinha, Instituto de Biologia, Universidade Federal Fluminense, Niterói, Brazil; ^4^Instituto de Microbiologia Paulo de Góes (IMPPG), Departamento de Virologia, Universidade Federal do Rio de Janeiro, Rio de Janeiro, Brazil; ^5^Instituto Fernandes Figueira (IFF), Fundação Oswaldo Cruz (Fiocruz), Rio de Janeiro, Brazil; ^6^Department of Biosciences, College of Science, Swansea University, Swansea, United Kingdom; ^7^Laboratório de Biologia e Taxonomia de Algas (LABIOTAL), Programa de Pós-graduação em Biodiversidade Neotropical, Instituto de Biociencias, Universidade Federal do Estado do Rio de Janeiro, Rio de Janeiro, Brazil; ^8^Bernhard Nocht Institute for Tropical Medicine, WHO Collaborating Centre for Arbovirus and Haemorrhagic Fever Reference and Research, Hamburg, Germany; ^9^Department of Molecular and Structural Biochemistry, North Carolina State University, Raleigh, NC, United States

**Keywords:** chikungunya, arbovirus, seaweed, antiviral, crude extracts

## Abstract

Chikungunya virus (CHIKV) infection is one of the most challenging re-emergent diseases caused by a virus, and with no specific antiviral treatment it has now become a major public health concern. In this investigation, 25 blood samples were collected from patients with characteristic CHIKV symptoms and submitted to a virus isolation protocol, which detected 3 CHIKV isolates. These samples were evaluated by sequencing for the characterization of the strains and any homology to viruses circulating in Brazil during a recent outbreak. These viruses were used for the development of antiviral assays. Subsequently, the inhibitory effects of seaweed extracts on CHIKV replication were studied. The marine species of algae tested were *Bryothamnion triquetrum, Caulerpa racemosa, Laurencia dendroidea, Osmundaria obtusiloba*, *Ulva fasciata*, and *Kappaphycus alvarezii*, all of which are found in different countries including Brazil. The results revealed high levels of CHIKV inhibition, including extracts of *O. obtusiloba* with inhibition values of 1.25 μg/mL and a selectivity index of 420. Viral inhibition was dependent on the time of addition of extract of *O. obtusiloba* to the infected cells, with the optimal inhibition occurring up to 16 h after infection. Neuron evaluations with *O. obtusiloba* were performed and demonstrated low toxicity, and in infected neurons we observed high inhibitory activity in a dose-dependent manner. These results indicate that the algal extracts may be promising novel candidates for the development of therapeutic agents against CHIKV infections.

## Introduction

First described in Tanzania in the African continent in 1952 and later definitively identified in Thailand in 1958, Chikungunya virus (CHIKV) has become a serious public health problem. CHIKV is a reemerging arbovirus infection, with significant human morbidity, and until now one million suspected cases have occurred worldwide (Burt et al., 2012; Presti et al., 2014; Brasil, 2018). CHIKV is mainly transmitted by *Aedes albopictus* and *Aedes aegypti*. Infection causes a self-limited febrile illness known as chikungunya fever with symptoms that include myalgia, fever, rash and debilitating joint symptoms such as persistent polyarthritis, which has been reported to last for many months or even years (Schilte et al., 2013). During a mild course of infection, symptoms usually appear after 4–7 days incubation and disappear after about 1 week from onset of symptoms (Grischott et al., 2016; Purpura et al., 2016).

Although CHIKV had not yet been considered a neurotropic virus, there is evidence now of its involvement as a cause of neurological diseases at different ages, although the maternal-child stage has been described as the main target in the manifestations of encephalopathy by this virus. Further studies are needed for the neurotropic correlation of CHIKV, although there is evidence of some probable neurological complications including encephalitis, myelopathy, peripheral neuropathy, melopoeia, and myopathy (Arpino et al., 2009; Chandak et al., 2009).

Arboviruses have symptoms that may be very difficult to differentiate because of their high overlapping characteristics, and thus clinical diagnosis has become a major challenge, mainly due to the current lack of effective methodologies for more accurate diagnosis. Some tests may be more decisive for CHIKV characterization, such as the reduction of platelet numbers and the presence of eruptions. However, the clinical signs and symptoms of CHIKV are indistinguishable from those of dengue fever, and both diseases are transmitted by *Aedes* mosquitoes (Rudolph et al., 2014). Therefore, steps should be taken to minimize misleading diagnosis during patient examination.

Arbovirus outbreaks, mainly of Zika virus (ZIKV), CHIKV, and dengue (DENV), have been frequently reported. In Thailand, CHIKV outbreaks were first documented in the early 1960s, and the latest outbreak was reported in 2008–2009 (Dash et al., 2007; Montagnier, 2010). Studies have shown that CHIKV was transmitted to Europe, in south-eastern France, between 2010 and 2014. However, in 2013, the first cases of autochthonous transmission in the French Caribbean were reported. Throughout 2018, the distribution of CHIKV in the Americas was also observed, reaching more than one million people (Leparc-Goffart et al., 2014; Powers, 2015).

The first autochthonous cases of CHIKV infection in Brazil were confirmed in 2014, in Oiapoque, Amapá (Rudolph et al., 2014). In 2017, there were 161,346 probable cases of CHIKV fever. In 2018, as of June 23, there were 53,089 probable cases of chikungunya fever in this country. Until this latter date, 11 deaths were confirmed from CHIKV while in the same period of 2017, 160 deaths were recorded (Brasil, 2018). To date, four CHIKV genotypes have been described, namely, East-Central-South African (ECSA), Asian, Indian Ocean, and West African (Weaver and Forrester, 2015). The Asian genotype was first detected in the Caribbean region in late 2013 and then spread throughout Central America. Nine months later, the first autochthonous cases in Brazil were detected in Oiapoque City, Amapá State and also in Feira de Santana, Bahia State, which are more than 2,000 Km from each other. Curiously, the ECSA genotype may be circulating in Feira de Santana and this has been shown to be from an individual who had recently returned from Angola and had a symptomatic contact in Feira de Santana (Nunes et al., 2015).

Chikungunya virus is an alphavirus of the Togaviridae family and has an 11.8 kb genome positive-sense single-stranded RNA. Alphavirus particles are enveloped, have a diameter of 70 nm, tend to be spherical (though slightly pleomorphic) and have an isometric nucleocapsid of 40 nm. The central region of the nucleocapsid has about 240 copies of the capsid protein that surrounds the viral genome. In addition, studies have shown that a number of host factors can be added to the nucleocapsid (Sokoloski et al., 2013). The lipid bilayer is strictly from the host and the source of the viral budding site (Strauss and Strauss, 1994; Lu and Kielian, 2000). Alphaviruses encode four non-structural proteins (nsp1, nsp2, nsp3, and nsp4), which are fundamental for viral genome replication, and also the structural proteins that include the capsid, and are related to the viral assembly process (Strauss and Strauss, 1994).

There are no vaccines or specific treatments available for the high morbidity rate caused by CHIKV. The most effective treatments are only symptomatic and use analgesics or anti-inflammatories. Therefore, new compounds are urgently required that can act on the CHIKV infection, reducing the high morbidity and mortality rates resulting from this virus. In this present study, natural products derived from marine algae have been tested against CHIKV as a new strategy since, previously, these extracts have been shown to have high activity against different microorganisms and also to be non-toxic for mammalian cells (Bourjot et al., 2012; Abdelnabi et al., 2015; Ching et al., 2015).

## Materials and Methods

### Seaweed Material and Extraction

The project obtained a permit for scientific purposes on 01/07/2012 at SISBIO/IBAMA number 3534 (VLT) and access to genetic heritage (register SISGEN – IBAMA) from the Universiade Federal Fluminense number A05E653 (VLT) in 01/11/2018.

The seaweeds were collected by snorkeling at a depth 1–3 m in various sites from the Brazilian coast. *Bryothamnion triquetrum* (S. G. Gmelin) M. Howe was collected at Atol das Rocas reef, Rio Grande do Norte State (lat. 03° 51′03′′, long. 33° 40′29′′), *Caulerpa racemosa* (Forsskål) J. Agardh was collected at Baía da Ribeira, Angra dos Reis, Rio de Janeiro State (lat. 22° 98′33′′, long. 44° 38′33′′), *Kappaphycus alvarezii* (Doty) Doty ex P. C. Silva was harvested from mariculture at Praia Grande, Paraty, Rio de Janeiro State (lat. 23° 16′15′′, long. 44° 34′48′′), *Laurencia dendroidea* J. Agardh was collected at Orla Bardot, Armação de Búzios, Rio de Janeiro State (lat. 22° 05′03′′, long. 41° 53′ 01′′), *Osmundaria obtusiloba* (C. Agardh) R. E. Norris was collected at Rasa Beach, Armação de Búzios, Rio de Janeiro State (lat. 22° 45′40′′, long. 41° 54′ 32′′) and *Ulva fasciata* Delile was collected at Itaipú Beach, Niterói, Rio de Janeiro State (lat. 22o 58′26′′, long. 43° 02′ 46′′).

The seaweeds were separated from sediments, epiphytes and other associated organisms, washed with sea water and air-dried (approximate temperature 28–30°C for 7–10 days) until the total evaporation of any water.

Air-dried seaweeds (approximately 100 g) were powdered and exhaustively extracted three times using different organic solvents (PA.) each time for 72 h. *Bryothamnion triquetrum* was extracted with dichloromethane, *Caulerpa racemosa* was extracted with acetone, *Kappaphycus alvarezii* was extracted with ethanol, *Laurencia dendroidea* was extracted with hexane, and *Osmundaria obtusiloba* and *Ulva fasciata* were extracted with ethanol.

The extracts were evaporated under reduced pressure, yielding crude extracts of each species (15–20 mg), of which 2–5 mg were used in tests against the CHIKV. The extracts were chosen according to their chemical composition, and by the presence of different classes of active substances (terpenes, sterols, fatty acids, polysaccharides, alkaloids, aromatic compounds) from these algae as indicated by previous studies (Santos et al., 2010; de Souza Barros et al., 2016; Lira et al., 2016). Analytical TLC was performed on Merck Kieselgel GF_254_ plates, spot detection was obtained by spraying with a 2% solution of Ce(SO4) in 2N H_2_S0_4_ followed by heating for 5 min at 150°C. The extracted compounds were examined by ^1^H NMR for detection of the majority of substances in each extract. ^1^H NMR was determined on a Varian-VNMRS apparatus at 300 and 500 MHz. Spectra were recorded in CDCl_3_ solutions using TMS as internal standard.

The thin layer chromatographic (TLC) used silica gel GF_254_ and various solvents systems as eluents and Nuclear Magnetic Resonance Proton (^1^H NMR) analyses demonstrated the majority presence of fatty acids and sterols in *B. triquetrum*, the caulerpin; a pigment bis-indole alkaloid from *C. racemosa;* the sterols, mainly cholesterol, from *K. alvarezii;* elatol, a halogenated sesquiterpene from *Laurencia dendroidea*; bromo-phenols from *Osmundaria obtusiloba*; and palmitic acid and other fatty acids from *U. fasciata.* All the products mentioned were obtained in previous studies (Rocha et al., 2007).

For the *in vitro* experiments the crude extracts were diluted in 100% DMSO and then added with culture medium to the final concentration of 0.01% DMSO.

### Cell Lines

VERO cells (African green monkey kidney) VERO-ATCCCCL81 were plated in Eagle’s minimum essential medium (MEM) (GIBCO) supplemented with 5% Fetal Bovine Serum (FBS). C6/36 mosquito cell line from *Aedes albopictus*, adapted to grow at 33 °C, was cultured in L-15 Medium (Leibovitz) supplemented with 0.3% tryptose phosphate broth, 0.02% glutamine, 1% MEM non-essential amino acids solution and 5% FBS. Purified cultures of retinal neurons were prepared according to earlier descriptions (Mejía-García and Paes-de-Carvalho, 2007). Retinas of 8-day-old chicken embryos were excised, incubated with 0.1% trypsin and then the cells were dissociated in medium with the help of a conical Pasteur pipette. Subsequently, the cells were seeded on 24 well plastic plates coated with poly-L-ornithine at a density of 830 cells/mm in BME containing 2.5% fetal calf serum, 100 U/ml penicillin, 100 mg/ml streptomycin and 2 mM glutamine and incubated at 37°C for 3 days in a 5% CO2 atmosphere. These purified cultures of retinal neurons were firstly described by Adler et al. (1984). In this work, they showed that almost 100% of the cells possess a neuronal identity using techniques such as phase-contrast microscopy, lectin staining and electron microscopy. After that, some groups have been working with this kind of culture to study different effects on neuronal cells, such as (Ferreira and Paes-de-Carvalho, 2001; Paes-de-Carvalho et al., 2003; Mejía-García et al., 2013) among others. More recently, Anccasi et al. (2013) demonstrated by immunocytochemistry assays that these cells are positive to Tuj1 (beta-tubulin III), which is a protein only found in neurons and considered as a neuronal marker, but negative to a glial marker 2M6.

### Virus Isolation

Between the months of March and April 2016, 25 samples of 5 mL venous whole blood were collected in a tube containing EDTA anticoagulant. Samples were taken from selected patients who had clear clinical symptoms of arbovirus infections, mainly joint symptoms and fever, and were treated at a hospital in Niteroi, RJ. The patients were interviewed and agreed to blood collection by signing the free and informed consent form, approved by the ethics committee with approved registration – CAAE: 61845416.0.0000.5289. After collection, all samples were submitted to RT-PCR analysis to determine if the patients had other viral infections, either Flavivirus or Alfavirus, and not only CHIKV. Samples were centrifuged for 5 min, and the leukocyte pellet was removed and added to 24-well plates at 90% confluence in VERO cells. After 24 h, the blood cells were removed from the VERO cell monolayer, fresh media was added, containing 5% FBS, and incubated in an atmosphere of 5% CO_2_ at 37°C for 3 to 5 days. The cell culture plates were evaluated daily in order to determine any cytopathic effect as a result of viral infection.

### RNA Detection by RT-qPCR and Virus Sequence

RNA from samples presenting cytopathic effects was extracted using the commercial kit QIAamp viral RNA mini (QIAGEN, Valencia, CA, United States) according to the manufacturer’s instructions. Isolation was confirmed by reverse transcription real-time PCR (RT-PCR) using primers targeting the structural polyprotein (forward TATCCTGACCATCCGACCCT/reverse GGCTCTTGTCCTTGCACTCT) and Superscript III One-Step RT PCR Kit (Invitrogen Carlsbad, CA, United States), according to the manufacturer’s instructions. Amplification was performed in the PeqStar (PeqLab, Erlangen, Germany). PCR conditions were as follows: 60°C for 1 min, 50°C for 45 min, 94°C for 2 min followed by 45 cycles of 95°C for 15 s, 55°C for 30 s and 68°C for 60 s.

The resulting amplicons were sequenced in the ABI 3730 genetic analyzer (Applied Biosystems) following the manufacturer’s protocol. Raw sequence data were aligned, edited and assembled using the Assembler tool Bioedit Sequence Aligner Editor. The identity was confirmed by using the Basic Local Alignment Search Tool (Blast) and compared to other CHIKV sequences. Phylogenetic tree was constructed using MEGA 7 program. The sequences were deposited in GenBank under accession numbers MK910738 (BRA/RJ/1F), MK910739 (BRA/RJ/18), and MK910740 (BRA/RJ/23). For the antiviral activity assays the strain with deposit number MK910739 was used.

### Plaque Reduction Assay

VERO cells were cultured in growth medium DMEM. The cells were then incubated with CHIKV for 2 h, washed with PBS and a mixture of 2% (w/v) carboxymethylcellulose (Sigma-Aldrich) and DMEM supplemented with 5% FCS, 5 mM L-glutamine and 0.20% sodium bicarbonate was added. Serial dilutions of compounds without overlap medium were done. Cells were fixed with 10% formaldehyde subsequently stained with 1% violet crystal. The infectious virus titer (PFU/ml) was determined using the following formula: plaque count × dilution factor × (1/inoculation volume).

### Antiviral Assay

Antiviral activity was evaluated using a virus plaque reduction assay. Vero cells and Neurons were grown in 24-well plates, as described above, and subsequently infected with MOI of 0.1 CHIKV in the absence or presence of different concentrations of the compounds. After 1 h of adsorption at 37°C, the residual inoculum was replaced by MEM containing 1% methyl-cellulose and the corresponding dose of each compound. Plaques were counted after 5–10 days of incubation at 37°C, at 5% CO_2_. Uninfected and treated neurons were incubated for 48 to 72 h, and the cells were evaluated daily for cytopathic effects and the culture supernatant was collected to determine the reduction of viral RNA production by RT-PCR. For VERO cells, the inhibitory concentration 50% (EC_50_) was calculated as the compound concentration reducing virus plaques by 50%. The Selective Index (SI) is derived from the relationship between the CC_50_ and the EC_50_ and reflects the potency and possible future selectivity for future drug development. All determinations were performed twice and each in triplicate.

### Time of Addition

VERO cells were infected with CHIKV at a MOI of 0.1 and incubated for 2 h. Afterward, the viruses were removed and the medium was replaced. In addition, 2.5 mg/mL of *C. racemosa* and *O. obtusiloba* crude extracts were added at different points of virus replication at 3, 2 or 1 h before infection, time 0 (immediately after virus added) or 1, 2, 4, 8, 12, 16, 20 or 24 h after infection. These cells were incubated in 5% CO_2_ atmosphere at 37°C for 72 h, and then viral replication was measured by plaque assay. Titration at all times was performed at the end of the experiment.

### Virucidal Effect

A CHIKV suspension containing 10^6^ PFU/mL was incubated with different concentrations of *C. racemosa*, *O. obtusiloba*, and *K. alvarezii* (2.5, 5 or 10 μg/mL) for 2 h at 37°C. The CHIKV suspension was also incubated with the same volume of solvent in the extracts (0.01% DMSO). Then, the samples were diluted in MEM and the remaining infectivity was titrated by plaque formation after 48 h. The important point about the extracts is that the sample dilution effectively reduced the drug concentration incubated with the cells by at least 200-fold to confirm that the titer reduction was only due to cell-free virion inactivation. The 50% relative virucidal effect defined inactivation compared to the controls.

### Statistical Analysis

The data were analyzed by the Tukey test or Dunnett test comparing all with the controls (ribavirin and DMSO) using the GraphPad Instat version 3 program. A *p*-value of <0.05 was considered statistically significant. The values of *p* < 0.05 and *p* < 0.01 are shown in the figures.

## Results

### RT-PCR of the Isolated Samples and Sequencing

**FIGURE 1:**
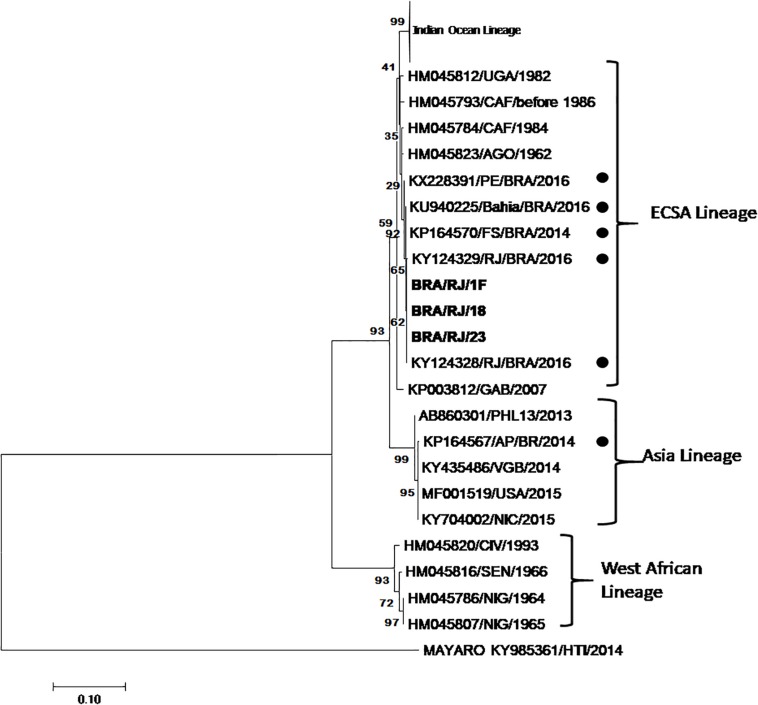
Phylogenetic tree was construct for partial non-structural protein (710 pb). Performed by using the Maximum Likelihood method based on the Kimura 2-parameter model. The analysis involved 69 nucleotide sequences. One fragment of sequences described in this studied is in bold, BRA/RJ/1F, BRA/RJ/18, and BRA/RJ/23 (GenBank access numbers MK910738; MK910739; MK910740). Dots mark the Brazilian isolates.

C6/36 mosquito cell line from *Aedes albopictus* was exposed to blood samples from patients suspected of infection by ZIKV or CHIKV and observed daily. At each passage in the C6/36 cells, 50 μL of the supernatant was removed and added into VERO cell cultures in which, at the fourth passage after the supernatant was added, the cytopathic effect was observed after 48 h. Of the 25 samples evaluated from patients who exhibited symptoms characteristic of CHIKV, such as fever, rash, muscular pains, joint pains, and headache, 3 presented cytopathic effects so that the culture supernatants were removed and analyzed by RT-PCR. All 3 samples were positive for CHIKV by PCR, but in order to confirm identity, nucleotide sequencing was performed to characterize the chikungunya strains. Phylogenetic Analysis confirmed the presence of the ECSA genotype in Rio de Janeiro. All viral infection experiments were performed with only one isolate as we determined in an initial experiment that there was no difference in the results obtained for each isolate. The isolates were closely related to strains previously detected in Bahia, Pernambuco and Rio de Janeiro (Figure 1).

### Cytotoxicity in VERO and Neuron Cells

**FIGURE 2:**
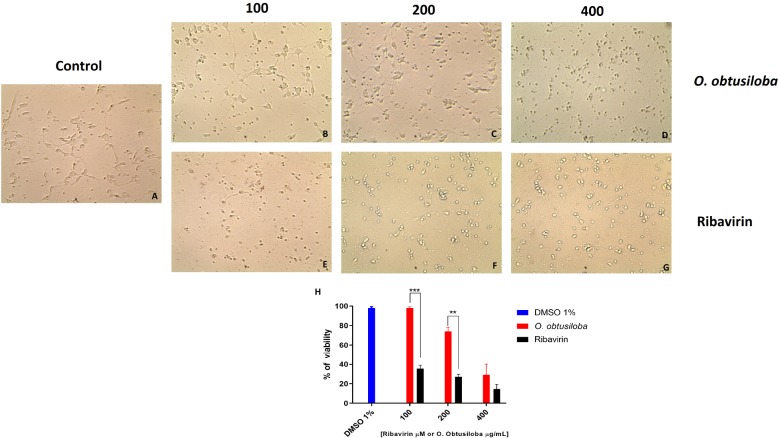
Cytotoxicity of *O. obtusiloba* extract and ribavirin in chick retina neurons in 4-day-old purified cultures. **(A)** Control culture. **(B,C,D)** Cultures incubated with *O. obtusiloba* at concentrations of **(B)** 100 μg/mL, **(C)** 200 μg/mL and **(D)** 400 μg/mL. **(E,F,G)** Cultures incubated with *ribavirin* at concentrations of **(E)** 100 μM, **(F)** 200 μM and **(G)** 400 μM. **(H)** Graphical representation showing the percentage of viability of treated cells with *O. obtusiloba* (blue bar) and percentage of viability of treated cells with ribavirin (dark bar). The results were evaluated by MTT assay and showed that the exposure to the *O. obtusiloba* extract presented low cytotoxicity maintaining high viability in concentrations up to 200 μg/mL. Error bars indicate the standard deviation and experiments were performed in triplicate. ^∗∗^*p* < 0.01; ^∗∗∗^*p* < 0.001 in Tukey test.

The cytotoxicity (CC_50_) of algae extracts in Vero and neuron cells was assessed by MTT [3-(4,5-dimethylthiazole-2-yl)-2,5-diphenyltetrazolium bromide] (Sigma-Aldrich), as previously described (Mosmann, 1983) using 10^5^ cells in 96-well plates. Vero cells were exposed to increasing concentrations of the compounds (50, 100, 200, 400, 800, and 1000 μg/mL) and incubated for 72 h to determine cell viability. The results in Table 1 show that in Vero cells the CC_50_ values for the extracts ranged from 178 to 732 μg/mL. In the neurons exposed to the *O. obtusiloba* extracts, there was low cytotoxicity with high viability maintained in concentrations up to 200 μg/mL. In contrast, ribavirin presented cytotoxicity from 100 μg/mL (Figure 2).

**TABLE 1:** Cytotoxicity (CC_50_) in Vero Cells, anti-CHIKV profile (EC_50_) and selectivity index (SI) of crude extracts from six seaweeds and ribavirin.

**Crude extracts**	**CC_50_^a^ (μg/mL)**	**EC_50_^b^ (μg/mL)**	***SI*^c^**
*C. racemosa*	732 ± 18.1	4.2 ± 0.83	174.2
*L. dendroidea*	178 ± 8.2	7.78 ± 1.1	22.9
*U. fasciata*	245 ± 11.6	18.9 ± 3.8	12.9
*O. obtusiloba*	525 ± 15.4	1.25 ± 0.36	420
*K. alvarezii*	423 ± 19.4	3.25 ± 0.9	130.1
*B. triquetrum*	400 ± 13.5	3.30 ± 0.82	121.2
Ribavirin	118 ± 5.88	1.73 ± 0.55	68

*Data represented as mean ± SD from three independent experiments. ^*a*^Concentration that reduced the cytotoxic concentration in Vero cells by 50% when compared to untreated controls. ^*b*^Concentration that reduced the CHIKV replication by 50% when compared to infected controls. ^*c*^Selectivity index was defined as the ratio between CC_50_ and EC_50_ and represents the safety for *in vitro* assays.*

### Inhibition of Chikungunya Virus by Marine Algae

**FIGURE 3:**
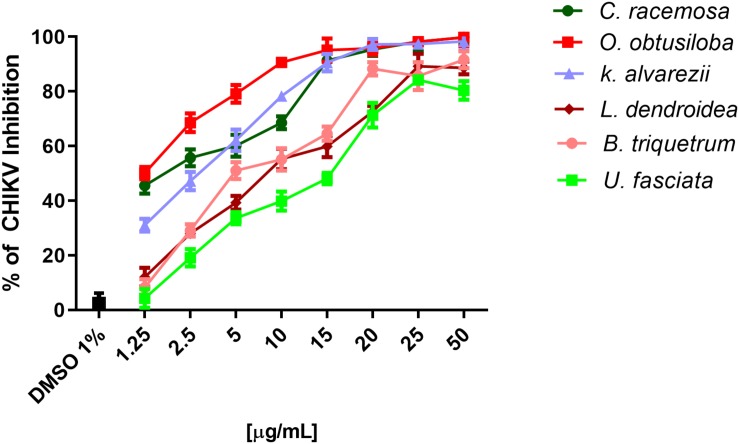
Effect of six seaweeds crude extracts on CHIKV replication. Vero cells were infected with CHIKV (10^6^ PFU/mL) at MOI of 0.1 and treated with different concentrations of the extracts, namely, 1.25, 2.5, 5, 10, 15, 20, 25, and 50 μM. The results were evaluated by plaque assay. Error bars indicate the standard deviation and experiments were performed in triplicate. DMSO used as solvent remained at the final concentration of 0.01% showing no activity on CHIKV replication.

**FIGURE 4:**
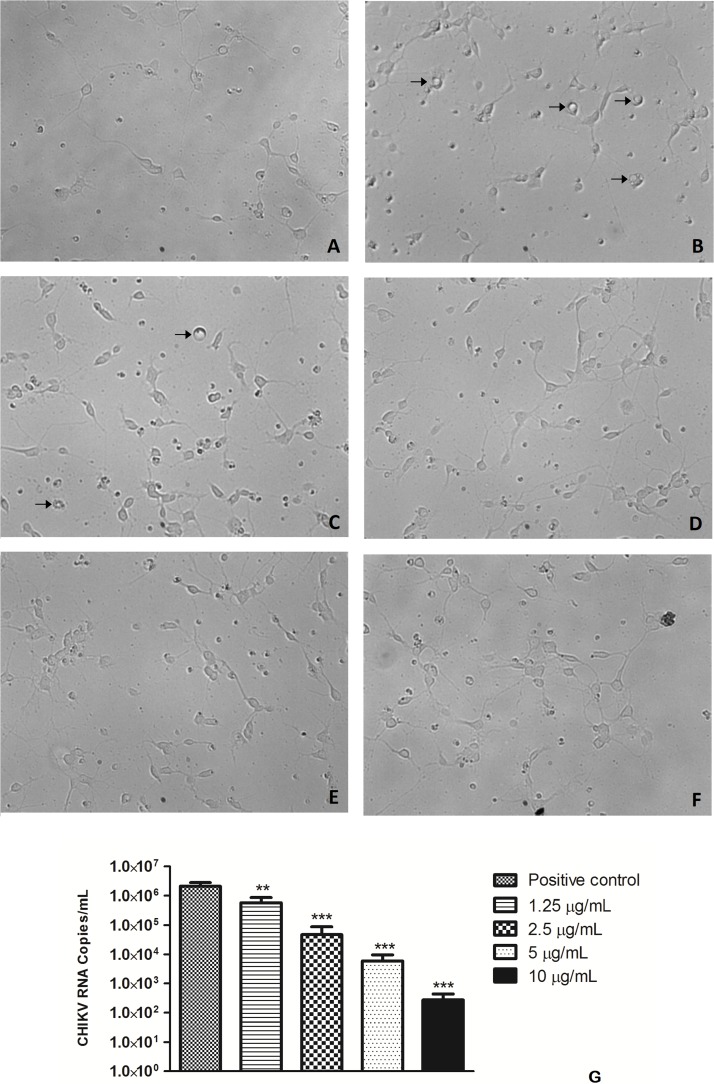
Effect of *O. obtusiloba* extract on CHIKV replication in chick retina neurons in 4-day-old purified cultures. **(A)** Uninfected culture with normal morphology. **(B)** CHIKV-infected culture presenting cytopathic effect (arrow). **(C)** CHIKV-infected culture incubated with 1.25 μg/mL of *O. obtusiloba* presenting cytopathic effect (arrow). **(D)** CHIKV-infected culture incubated with 2.5 μg/mL of *O. obtusiloba* with normal morphology. **(E)** CHIKV-infected culture incubated with 5 μg/mL of *O. obtusiloba* with normal morphology. **(F)** CHIKV-infected culture incubated with 10 μg/mL of *O. obtusiloba* with normal morphology. Chick retina neurons were infected with CHIKV (10^6^ PFU/mL) at MOI of 0.1. **(G)** Graphical representation showing the effect of *O. obtusiloba* extract at concentrations of 1.25, 2.5, 5, and 10 μg/mL on CHIKV genomic RNA by quantitative PCR. The results showed that all treatments with the extract showed a significant reduction of RNA copies. Error bars indicate the standard deviation and experiments were performed in triplicate. ^∗∗^*p* < 0.01; ^∗∗∗^*p* < 0.001 in Tukey test.

To evaluate the potential of the extracts to inhibit CHIKV replication, Vero cells and neurons were infected in 96-well plates with 0.1 CHIKV MOI, incubated for 1 h for viral adsorption and, subsequently, treated with increasing concentrations of algae extracts. Figure 3 shows that all the extracts inhibited the replication of CHIKV in a dose-dependent manner. *C. racemosa*, *O. obtusiloba* and *K. alvarezii* inhibited 30 to 98% of CHIKV replication at concentrations from 1.25 to 50 μg/ml. However, the *O. obtusiloba* extract had the highest EC_50_ value (viral inhibition) at 1.25 μg/mL and had a CC_50_ of 525 μg/mL ± 3.11, generating a selectivity index (SI) of 420. In contrast, *C. racemosa* and *K. alvarezii* had lower SIs at 174.2 and 130.1, respectively. Ribavirin, which was used as a control, had an EC_50_ value of 1.73 ± 0.55 μg/mL and was less potent than *O. obtusiloba* extract in inhibiting CHIKV replication (Table 1). In infected neurons treated with *O. obtusiloba* extract, there was a significant reduction in the production of copies of viral RNA, demonstrating a strong inhibitory effect of the replication of CHIKV by these compounds (Figure 4).

### Virucidal Effect

**FIGURE 5:**
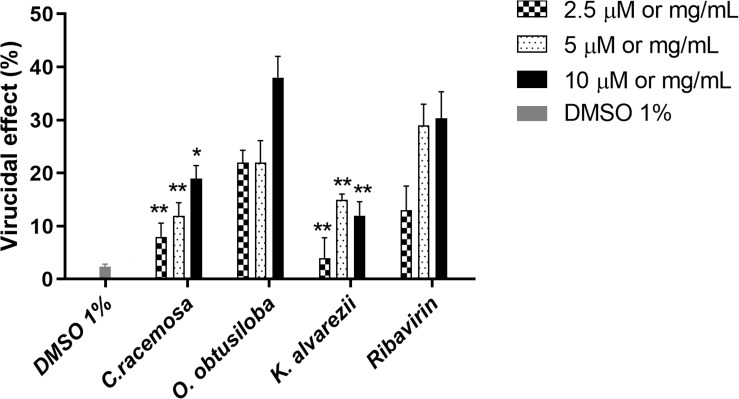
Effect of C. racemosa, O. obtusiloba and K. alvarezii crude extracts on infectivity of CHIKV. To assess potential virucidal activity, we incubated stock CHIKV [CHIKV (106 PFU/mL) at MOI of 0.1] directly with each compound separately at different concentrations for 1 h or with vehicle/DMEM, after which we determined virus titers by standard plaque assays in Vero cells. Experiments were performed independently in triplicate with duplicate plate assays. Error bars indicate the standard deviation and experiments were performed in triplicate. ^∗^*p* < 0.05; ^∗∗^*p* < 0.01 in Dunnett test vs. control (Ribavirin). DMSO used as solvent remained at the final concentration of 0.01%, showing no virucidal activity on CHIKV.

The extracts exhibiting the highest inhibition profiles of CHIKV were selected to test for virucidal effects. To this end, the virus was subjected to three concentrations of the compounds (10, 5, and 2.5 μg/ml) for 2 h at 37°C. Figure 5 shows that the extracts tested had limited virucidal potentials. However, *O. obtusiloba* was able to inhibit about 40% of CHIKV replication in the highest concentration of extract used (10 μg/mL). In contrast, Ribavirin used as a control at the same concentration, only inhibited 29% of the production of viral plaques. DMSO used as solvent remained at the final concentration of 0.01%, showing no virucidal activity on CHIKV.

### Time of Drug Addition

**FIGURE 6:**
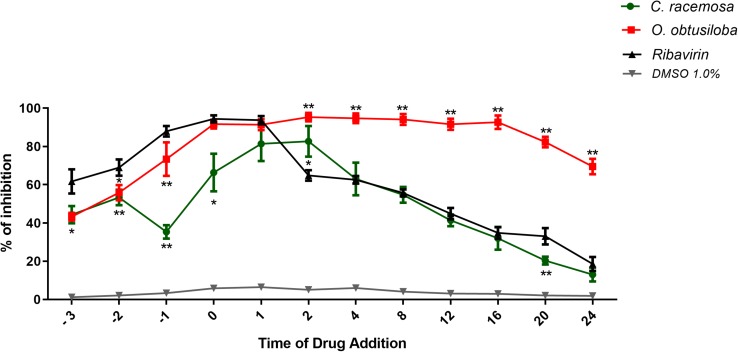
Time of drug addiction studies with *C. racemosa*, *O. obtusiloba*, and *K. alvarezii* crude extracts on CHIKV replication. Vero cells were infected with CHIKV (10^6^ PFU/mL) on MOI of 0.1 following each treatment (before the infection, –3, –2, and –1 h; during the infection, 0h; and after the infection, +1, +2, +4, +8, +12, +16, +20, and +24 h), the results were evaluated by plaque assay. Experiments were performed in triplicate. Ribavirin (5 μM) was used as a positive control. ^∗^*p* < 0.05; ^∗∗^*p* < 0.01 in Dunnett test vs. control (Ribavirin). DMSO used as solvent remained at the final concentration of 0.01% showing no activity on CHIKV replication.

In order to investigate the mechanism of action of the tested compounds, a time of addition assay was performed using extracts of *C. racemosa* or *O. obtusiloba*, which showed more promising results. For this, VERO cells were treated with *C. racemosa* or *O. obtusiloba* using the antiviral drug Ribavirin, as a control. Figure 6 shows that when *C. racemosa* or *O. obtusiloba* extracts were added prior to infection of the cells, they produced a pretreatment effect inhibiting about 40% of viral replication. However, when added at time 0, we observed an effect greater than 60% for *C. racemosa* and above 90% for *O. obtusiloba* and Ribavirin. Although Ribavirin was more effective in inhibiting the virus than seaweed extracts between −3 and −1 h, its effect decreased steadily within 2 h after infection, greatly reducing its effects on post infection. In contrast, the effect of *O. obtusiloba* persisted for at least 16 h after infection, slowly reducing to 60% at 20 and 24 h and was significantly greater (*p* < 0.01) than the Ribavirin controls.

## Discussion

There are no effective antivirals or vaccines against CHIKV, and as a result this infection has had a significant public health impact, particularly in Brazil and the Americas between 2014 and 2015 (Tan et al., 2018), affecting large numbers of people (Ching et al., 2015; Ahmadi et al., 2016). Numerous studies have been undertaken to discover effective drugs to control this infection, and as a result certain drugs with inhibitory potential for the replication of CHIKV replication, such as Ribavirin, Favipiravir and chloroquine, were developed (Delang et al., 2014; Lani et al., 2015; Ahmadi et al., 2016; Varghese et al., 2016). In the present study, *O. obtusiloba* showed promising results in inhibiting the replication of CHIKV compared to Ribavirin, which has been described as a potential choice for the treatment of CHIKV infection and was used as a control for our studies (Rothan et al., 2015).

The experiments evaluated blood samples of patients suspected of CHIKV infection in the second half of 2016 and who did not travel during this period. This allowed the identification of patients who had been infected by the CHIKV that was circulating in Rio de Janeiro. Samples were collected from 25 patients from Rio de Janeiro who had characteristic chikungunya symptoms and, after extensive attempts, successfully isolated the CHIKV from three patients. RT-PCR and sequencing confirmed that these CHIKV were the same as those in circulation in Brazil and that were initially identified in Bahia to later migrate to Rio de Janeiro (Nunes et al., 2015). This CHIKV were used for *in vitro* studies to test the inhibition of viral replication using seaweed extracts.

Marine organisms, including algae, have undergone many demographic studies as well as analyses of their antimicrobial, antifungal, antiviral, anti-inflammatory activities, and are sources of new therapeutic agents. More recently, the promising antiviral potential of algal derivatives has been targeted in pharmaceutical research, especially for HIV infections (Cirne-Santos et al., 2008; Ahmadi et al., 2015; Pérez et al., 2016).

In the present study, the algal extracts studied had low cytotoxicity in VERO cells with CC_50_ at concentrations higher than 170 μg/mL. The extracts of the algae, *C. racemosa* and *O. obtusiloba*, clearly showed particularly promising results with CC_50_ of 732 and 525 μg/mL, respectively. In relation to the inhibitory effect on CHIKV replication, the EC_50_ of *O. obtusiloba* extract was 1.25 μg/mL, considerably lower than the result obtained with the *C. racemosa* extract, which was 4.2 μg/mL. Comparing the SI levels of both (*C. racemosa* of 174.2 and *O. obtusiloba* of 420) also confirms their potential as possible candidates for further investigation in the discovery of novel anti-CHIKV drugs. Taking into account the inhibitory effects of Ribavirin, which has been inserted in several studies as a control and given results similar to those presented by the extracts, we can see a strong potential for further studies of the mechanism of action as well as the determination of the respective active compounds present in algae.

Some evidence of compounds derived from algae has been well described in the literature, showing significant effects on various viruses such as *Caulerpa racemosa* extract, where alkaloids and terpenoids are found, and in the acetonic extract the main component is caulerpine, which has antiviral activity against HSV-1 (Macedo et al., 2012; Pinto et al., 2012). Antiviral activity against HSV-1 and HSV-2 has also been described for glycolipids extracted from *O. obtusiloba* with EC_50_ values of 42 μg/mL and 12 μg/mL, respectively (de Souza et al., 2012). *O. obtusiloba* ethanolic extract also showed potent antiviral activity against ZIKV (EC_50_ = 1.82) (Cirne-Santos Claudio et al., 2018). De Alencar et al. (2016) showed that the 70% EtOH was the most effective solvent for extracting phenolic compounds from red seaweeds when compared to hexane, and *O. obtusiloba* EtOH extract presented high antioxidant activity. Seven substances were identified in Brazilian *O. obtusiloba*: three sulfated bromophenols, two bromophenols, one sterol and one glyceride (Carvalho et al., 2006). Given that *O. obtusiloba* ethanolic extract is rich in bromophenols, it is possible that these compounds may be responsible for the inhibitory effects of CHIKV replication.

The extract-tested compounds also had low virucidal potentials (Figure 5), with only *O. obtusiloba* resulting in about 40% virucidal activity at 10 μg/mL, although it was superior to the effect of the same concentration of Ribavirin that was used as a control. The study of compounds with this activity is highly relevant because virucidal compounds are chemicals that attack and inactivate viral particles outside the cell (virions), although it is possible that damage to the viral structure occurs (Galabov, 2007).

Several studies have also demonstrated the ability of CHIKV to infect neurons and glial cells with associated neurological complications, suggesting the neurotropic nature of the virus (Chandak et al., 2009; Das et al., 2010; Dhanwani et al., 2012). The results demonstrated that the CHIKV cytopathic effects on infected neurons can be reduced by treatment with *O. obtusiloba* (Figure 4). Increasing concentrations of *O. obtusiloba* have higher antiviral activity so that treatment with this compound should be more widely studied, as this may contribute to the reduction of morbidity and mortality of the clinical conditions such as encephalopathies and bone and joint disorders related to CHIKV. Thus, the observation of the reduction in viral RNA production demonstrated by O. obtusiloba tells us the important role of this compound in inhibiting CHIKV replication not only in summer cells but also in primary neurons (Figure 4).

Finally, the virus replication inhibition assays by varying the time of extract addition (Figure 6) showed that the addition of *O. obtusiloba* at time zero, at the same time as the virus, inhibited viral replication at a rate greater than 80%. This effect was maintained even if the extracts were added up to 16 h after CHIKV infection. There was a small decline in the inhibitory effect after this time, but the extract still inhibited CHIKV replication above 60% if the compound was added within 24 h post-infection. This demonstrates a protective effect even after prolonged contact of the VERO cells with the virus. Pretreatment with these same algal compounds exhibits inhibition of viral replication (approximately 40%), emphasizing the possibility of a virucidal and somewhat protective effect. Importantly, as observed in the time of drug addiction assays, the *O. obtusiloba* extract inhibited viral replication for a longer time than the Ribavirin used as control and also maintains its inhibitory effect for long periods post-infection. This may be vital in many clinical settings where the diagnosis takes some time.

Considering the promising results of the *O. obtusiloba* extract and the previous work of our group demonstrating the low toxicity of this extract administered orally in BALB/c mice (Barros et al., 2018), the extract of this alga becomes a good candidate for further studies. In addition, the possibility of oral treatment with the extract, or the active ingredient isolated from the extract, can also be tested, as was done with dolabelladienetriol (20 mg/Kg/dose; twice a day) against HSV-1 in BALB/c (Garrido et al., 2017).

## Data Availability Statement

The raw data supporting the conclusion of this manuscript will be made available by the authors, without undue reservation, to any qualified researcher.

## Ethics Statement

This project was submitted to the research ethics committee of ASSOCIACAO SALGADO DE OLIVEIRA DE EDUCACAO E CULTURA and obtained the registration approval – CAAE: 61845416.0.0000.5289.

## Author Contributions

CC-S, CB, KY, GM, and CN performed the experiments and wrote the manuscript. RA, ZV, and JS-C worked on the implementation of methodologies and data review. VT worked on the supply of natural product. NR worked on the revision of English and in writing. DF and IP worked as job coordinators.

## Conflict of Interest

The authors declare that the research was conducted in the absence of any commercial or financial relationships that could be construed as a potential conflict of interest.
